# Retinitis Pigmentosa: Pathogenesis, Diagnostic Findings, and Treatment

**DOI:** 10.7759/cureus.48006

**Published:** 2023-10-30

**Authors:** Saakshi P Kamde, Anil Anjankar

**Affiliations:** 1 Forensic Medicine, Jawaharlal Nehru Medical College, Datta Meghe Institute of Higher Education and Research, Wardha, IND

**Keywords:** retinitis pigmentosa (rp), retinal dystrophy, retinal diseases, diabetic retinopathy, fundus examination, treatment, gene therapy, cell therapy, therapeutic landscape

## Abstract

Retinitis Pigmentosa (RP) is an inherited retinal dystrophy (IRD) that causes progressive visual loss. Patients suffering from RP have a substantial influence on their everyday activities, social contacts, and jobs, lowering their quality of life. Frequent referral delays, as well as the lack of a standard therapy for the majority of patients, contribute to the significant unmet demand for RP. Any retinal injury has the potential to result in total blindness and visual impairment. Despite the fact that there is no cure for RP, people can manage it using rehabilitation programs and low-vision gadgets. The purpose of this research is to characterize the expanding treatment landscape for RP, as well as the justification for advanced therapy medicinal products (ATMPs).

Vitamin A supplements can help prevent the sluggish visual loss caused by a prevalent kind of RP. The presence of visual purple in the rods and the underlying vascular choroid causes the retina to look purplish red. The major portion of the retina damaged is the rod photoreceptor electric cell; the development of diverse diseases is progressive. Because of the retina's accessibility, immunological privilege, and compartmentalization, hereditary retinal diseases are amenable to cell and gene therapy. Therapeutic techniques that attempt to rescue photoreceptors (gene therapies) require the existence of non-functional target cells, but other therapies (cell therapies) do not require the presence of live photoreceptors. To provide successful therapy choices for RP patients at all disease phases, the development pipeline must be continually diversified and advanced, as well as ongoing efforts to encourage early patient identification and quick diagnosis. Future research will focus on avoiding vision loss in genetic eye illnesses and assisting patients in regaining their eyesight. Retinal implants, cell therapies, supplementary medications, and gene therapies may become common treatments for reducing vision loss in the future.

## Introduction and background

Retinitis Pigmentosa (RP) is a hereditary retinal dystrophy that causes progressive visual loss. The most prevalent inherited retinal dystrophy (IRD) is RP, which affects over 1.5 million people globally. RP is classified into two types: syndromic and non-syndromic. Non-syndromic RP begins as night blindness, develops into vision loss, and eventually falls into the peripheral vision range. Retinal dystrophies are defined depending on the location of the retina, the kind of cells damaged (rods, cones, or both), the retinal pigment epithelium (RPE), the inner retina, and whether the progression is stagnant or progressive. RP is a degenerative condition that causes individuals to lose their night vision first, followed by decreased visual acuity and a gradual narrowing of the visual field. By the age of 40, the majority of patients are legally blind. Increased difficulty completing everyday tasks and decreased autonomy are connected with worsening symptoms. Anxiety and depression levels are higher, social isolation is greater, and overall quality of life is lower. As a result, RP has a severe impact on all aspects of the patient's life, imposing a huge cost on patients, families and caregivers, and society.

Progressive dystrophy only related to cones is called cone dystrophy. Only one copy of a disease allele is required for a person to be susceptible to expressing the phenotype in autosomal dominant (AD) inheritance. Two copies of a disease allele are necessary for an individual to be at risk of manifesting the phenotype in autosomal recessive (AR) inheritance. For an X-linked dominant disease (XD) in the AD inheritance, just one copy of a disease allele on the X chromosome is necessary. For a person with two X chromosomes (a female) to have an X-linked recessive disease (XR), two copies of a disease allele on the X chromosome are necessary. This is similar to AR inheritance. The electric response of the light is delayed or received in less amount in RP, as the result is given by electroretinogram (ERG) with the characteristic resemblance of 'bone spicules' in the retina [1]. Usher syndrome is the most common form of syndromic RP, accompanied by loss of hearing. In non-syndromic RP, the histology findings show dilution of an outer nuclear layer as rods and cones photoreceptor cells die, an inherited retinal dystrophy. The brain interprets vision as rod and cone photoreceptors in the retina transform light into electrical impulses. Typically, weak light affects rods, resulting in night vision and peripheral vision degeneration. When cones are compromised in RP, the symptoms are central vision dimensions, color perception, and visual acuity. Genetic factors are important since this illness is caused by mutations in molecular genes. Tunnel vision is a condition in which the visual field compresses continually until just a small fraction of the center vision remains. [2-4]. The RPE performs the primary job of maintaining the photoreceptor layer. RP can be X-linked or recessive, dominant, or part of another condition, depending on the kind of molecular variant present. Recognizing the indicators of RP at the early stage is critical for effective medical care, as it allows for optimal management. In contrast to macular degeneration in RP, central vision is retained, but night vision and peripheral vision are lost [5,6].

The study delves into the genetic heterogeneity of the disease, diagnostic methods such as electroretinography (ERG) and optical coherence tomography (OCT), and potential treatments, including gene therapy, stem cell therapy, and vitamin supplements.

## Review

Methodology

**Figure 1:**
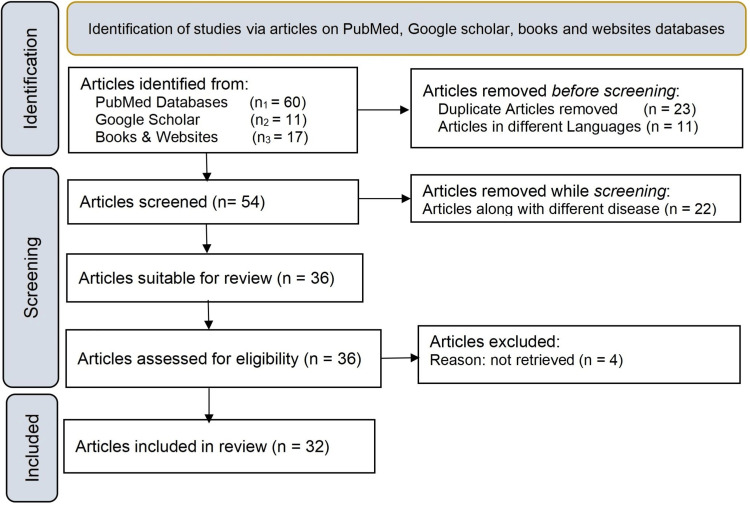
PRISMA methodology used in the study PRISMA: Preferred Reporting Items for Systematic Reviews and Meta-Analyses

This is a comprehensive review article that focuses on a rare eye disease of the retina known as RP and describes the pathogenesis, symptoms, diagnosis, and treatment. Numerous peer-reviewed journals and research articles, in addition to articles from Google Scholar and PubMed were used in this comprehensive review. Keywords used for the study are RP, visual impairment, retinal dystrophy, treatment, gene therapy, cell therapy, and therapeutic landscape. Studies from the previous 15 years were comprised and 32 references were included in this review article, which comprises accessing websites, articles from peer-reviewed journals, and medical books. The preferred report items for systematic reviews and meta-analyses (PRISMA) method used for the study is depicted in Figure 1.

The overall results of generic reporting aspects of reviews applicable to this systematic review are presented in Table 1. Search terms, years searched, inclusion or exclusion criteria, description of intervention, and categories of studies included were characteristics that were given by all reviews (more than 80%). Number of 34 reviews reported irrelevant in terms of duplication and other language of selection criteria. Infrequently reported items for results included using the PRISMA diagram (59 percent) and a description of other diseases along with the intended study is (43 percent).

**Table 1:** Reporting of articles searches, study selection, and results in systematic reviews

Reporting Item	Total N=32 n (%)
Search	
Search terms were described or referred to else where	30(93)
Multiple databases were searched	25(79)
Years searched were described	26(81)
Multiple languages were included in search	11(34)
Selection	
Inclusion or exclusion criteria were stated	31(96)
Interventions/exposures were described	32(100)
Reasons for exclusion were described	22(68)
Result	
The number of studies included and excluded was used	26(81)
Reviews that conducted a meta-analysis	30(93)

Pathogenesis

The majority of patients have symptoms of diminished peripheral vision on both an upper and lower visual field, as well as night vision loss. The dark pigmented spots are the pigments of melanin. Deoxyribonucleic acid (DNA) testing, visual field testing (VFT), ocular coherence tomography (OCT), and electroretinography (ERG) are some supportive testing methods used to determine the gene accountable for a particular type of RP called an inherited retinal dystrophy. Night blindness and peripheral vision are caused by rod cell degeneration. The eye is a non-digital camera with many elements, such as the lens, retina, and cornea. Any injury to these structures might lead to visual loss [7,8]. Cyclic guanosine monophosphate (cGMP) is greatly abundant in photoreceptor cells in the dark. This cGMP causes the entrance of positive ions such as sodium and calcium, resulting in depolarization. Depolarization causes glutamate to be released in the dark. The cell hyperpolarizes as the photoreceptor moves into the light. Light enters the eye, travels to the photoreceptors, and triggers a conformational change in opsin, a unique protein. This modification stimulates a G-protein known as transducin, which subsequently activates a protein known as phosphodiesterase (PDE). The major purpose of the phosphodiesterase is to convert cGMP into 5-GMP, the inactivated form of cGMP. The cGMP-dependent cell may be a therapeutic target for hereditary photoreceptor degeneration. When exposed to light, there is a drop in the quantity of cGMP, which causes the ion channels to close and inhibits positive ions from entering. The transfer of an electric signal from the retina to the processing center located in the occipital lobe of the cerebral cortex alters the potential of photoreceptor cells. Multiple genes that get mutated produce multiple defective proteins, resulting in the illness RP. It is a hereditary disorder that disrupts the normal physiology of phototransduction, that is, the conversion of light into a change in the electrical potential across the cell membrane. AD-inherited RP is caused by mutations in pre-mRNA splicing. The X-linked RP is caused by mutations in the RPGR and RP2 genes at their respective loci. Multiple mutations result in photoreceptor cells degenerating [9-11].

RP is mostly caused by mutations in numerous rod-specific genes, which then cause cone degeneration and alterations in the retinal pigment epithelium and ganglion cells. The fundus examination reveals the loss in retinal blood vessels in addition to the peripheral bone-spicule deposits. ERG is the prosperous diagnostic standard, showing the reduction in the rod and the cone response that are united with a delay in their timing. The visual field (VF) loss is a crucial indicator since it shows the disease's course and the effectiveness of treatment, ranging from patchy loss of the peripheral visual region to tunnel vision, ring scotoma, and eventually leading to total blindness. With the patterned advance of retinal degeneration in disease and the development of effective medical care of retinal and choroidal vasculature the imaging information from the high-resolution, optical coherence tomography angiography (OCTA), a valuable imaging technique, has become necessary. The lyonization is termed as turned off or inactivated development of one of the X chromosomes during development; the genes present on that chromosome are usually inactivated. To date, around 260 hereditary causal genes of genetically heterogeneous blinding diseases have been identified. The photoreceptor genetic modifications lead to the secondary degeneration of cones and the primary degeneration of rods. Rod photoreceptors begin to deteriorate in blinding disorderliness, progressing to cone photoreceptor death [12,13].

The causes of vision loss are affected retinal ganglion cells (RGC) and retinal pigment epithelium (RPE), changed inner retinal vascular supply, and damage to the outer retina as a result of these alterations. The microglias accumulated in the retina's plexiform layers are principally responsible for the continual monitoring of microenvironmental homeostasis. The movement of various photoreceptor triggers quickly degenerates microglia, which finally destroys the layers and transforms into phagocytes. In addition to phagocytosis, a considerable number of pro-inflammatory neurotoxic substances are secreted when interacting with invading blood cells. Other variables, such as hemodynamics and retinal vasculature, are linked to RP. The parasympathetic and sympathetic nervous systems control the choroidal tissue, whereas the retinal blood vessels can be regulated. The trigger for blood vessel autoregulation might be hypoxic or hyperoxic, which helps maintain retinal homeostasis. Vascular malfunction, rather than the presence of photoreceptors in the retina, may be related to mortality in RP [14]. According to histology, the primary cause of the illness is a deficiency in the rod and cone photoreceptors. According to pathological findings of an enucleated eye with autosomal recessive RP, two types of pigmented cells were observed in the retina: macrophage-like cells that contain melanin and veritable RPE cells that are transmigrating away from the retinal pigment epithelium layer. Photoreceptor involvement was assumed to be a reactionary response to photoreceptor injury. The outside segments, like the inner component, are poorly structured, truncated, or missing in autosomal dominant RP. The histopathology findings in RP are the perinuclear cytoplasmic membranous swirls and Inclusion bodies. The toxic level of cyclic guanosine monophosphate is raised due to a defect in cGMP phosphodiesterase [15].

When RP is advanced, there are no distinguishing features of rod and cone degeneration. The boundary that separates RP from congenital retinal sightlessness is also blurry. Congenital retinal vision impairment is difficult to identify since the ophthalmologist does not know how early and severe the visual loss must have happened in childhood. The loss of photoreceptors is measured by the ERG amplitudes and quantitative measures. The optic nerve atrophy followed by arteriolar attenuation and the changes in the pigment are the characteristics of RP. The particular genetic defect determines if rod cells are involved or the cone cells. The most common being the rod cell photoreceptor defect, cone cell dystrophy is rarely present. More than 80 genetic abnormalities and various other inheritance patterns have been identified. In the Usher syndrome, visual decline advances more rapidly than sensorineural deafness does. External ophthalmoplegia and heart block are also symptoms of Kearns-Sayre syndrome. Mental impairment is linked to the Bardet-Biedl and Laurence-Moon disorders. There is loss and breakdown of the photoreceptor cell. Arteriolar attenuation, retinal pigmentary alterations (either hypopigmentation or hyperpigmentation in the form of bone spicules and pigment clumping), and waxy disc pallor are the typical clinical trials of RP. The atrophy of the optic disc and the optical field failure are also characteristics of open-angle glaucoma. In the pathogenesis of the open-angle glaucoma (OAG), there are specific genetic variants and elevated intraocular pressure. There is the same cone vulnerability in the pathways of RP and OAG, which includes S and M cone loss. There is a similarity between the vascular dysfunction in OAG and the reduction in the blood flow in RP [16,17].

The retina's thin covering on the back of the eye is the primary source of vision and acceptance of a light beam. The retinal cells are neuroectoderm, the early embryonic ectoderm component that gives rise to the central and peripheral nervous systems. They are divided into seven layers and contain six different kinds of neuronal cells. The layers are the retinal pigment epithelium, photoreceptor cell layer, inner plexiform, nuclear region, and ganglion cell region. The role of the ganglion cell layer cells is to send visual signals to the optic nerve. The photoreceptor cell layer comprises the retina's light-excitable neurons. Cone and rod cell nuclei with synaptic connections are found in the outer nuclear and outer plexiform regions [18]. The retinal pigment epithelium is the last area that lacks neuronal cells; it promotes the preservation and growth of the photoreceptor cell layer, and the effect is mitigated by soaking up additional light. Several genes become the source of the disease of the same phenotype because of genetic heterogeneity. Phenotypic heterogeneity is seen when a particular mutation happens in the identical gene, which is a different disease; for example in autosomal dominant RP, the mutation in the rhodopsin produces autosomal recessive RP. Despite its intricacy, finding RP genes and mutations has advanced significantly. The allelic heterogeneity has been seen in cases where various diseases produce mutations in each gene. Syndromic and non-syndromic are the two types of RP. The identified 56 gene mutations become the source of non-syndromic RP. The mutated 23 genes are the AR and AD RP contains 36 mutated genes and X-linked RP has three mutated genes. Ushers syndrome is caused in the case of systemic form, with mutation observed in the 12 genes and Bardiet-Biedl syndrome is seen when the mutation occurs in 17 genes [19]. The places where the RP is disseminated include the macula, fovea, mid-periphery of the retina, and the cardinal retina. The disease's clinical symptoms include night blindness, or both night blindness and a progressive loss of peripheral vision known as "tunnel vision," which leads to total blindness in maturity. Numerous disease-causing mutations have been identified in the genes that cause RP in an individual. The discovery of genes and mutations has simplified the etiology of AD RP. The genes involved in AD RP and their functional roles are depicted in Table 2 (Adapted from [20, 21]). The genetic mutation in the DNA and also in the rods and cone cells are the major cause of RP. Rod and cone cell dystrophy are the other side problems in patients suffering from RP [22].

**Table 2:** Genes mutation causes autosomal dominant retinitis pigmentosa and functional role. BEST1: bestrophin 1; CA4: carbonic anhydrase 4; CRX: cone-rod homeobox protein; FSCN2: fascin actin-bundling protein 2; GUCA1B: guanylate cyclase activator 1B; HK1: hexokinase 1; IMPDH1: inosine monophosphate dehydrogenase 1; KLHL7: kelch like family member 7; NR2E3: nuclear receptor subfamily 2 group E member 3; NRL: neural retina leucine zipper; PRPF3: pre-mRNA processing factor 3; PRPF4: pre-mRNA processing factor 4; PRPF6: pre-mRNA processing factor 6; PRPF8: pre-mRNA processing factor 8; PRPF31: pre-mRNA processing factor 31; PRPH2: peripherin 2; RDH12: retinol dehydrogenase 12; RHO: rhodopsin; ROM1: retinal outer segment membrane protein 1; RP1: RP1 axonemal microtubule associated; RP9: RP9 Pre-MRNA Splicing Factor; RPE65: retinal pigment epithelium-specific 65 kD protein; SEMA4A: semaphorin 4A; SNRNP200: small nuclear ribonucleoprotein U5 subunit 200; TOPORS: TOP1 binding arginine/serine rich protein, E3 ubiquitin ligase

Sr. No.	Symbol	Chromosome	Functional Role of Gene/protein
1	BEST1	11q12.3	Visual cycle and chloride channel
2	CA4	17q23.2	Inherited Retinal abnormalities of bicarbonate transport
3	CRX	19q13.32	Transcription factor
4	FSCN2	17q25.3	A mutation results in one form of autosomal dominant rp and macular degeneration
5	GUCA1B	6p21.1	Activates photoreceptor guanylate cyclases. Mutations in this gene can cause a form of retinitis pigmentosa
6	HK1	10q22.1	Glucose metabolism, and hemolytic anemia due to hexokinase deficiency
7	IMPDH1	7q32.1	Defects in this gene are a cause of retinitis pigmentosa type 10
8	KLHL7	7p15.3	May be involved in protein degradation
9	NR2E3	15q23	A ligand-dependent transcription factor.
10	NRL	14q11.2	Transcription factor of the maf subfamily
11	PRPF3	1q21.2	The removal pre-mRNAs occurs on complexes called spliceosomes
12	PRPF4	9q32	A regulator of chemoresistance in human ovarian cancer
13	PRPF6	20q13.33	Providing a link between transcriptional activation and splicing
14	PRPF8	17p13.3	Function in protein-protein interactions
15	PRPF31	19q13.42	A component of the spliceosome complex and is one of several retinitis pigmentosa-causing genes
16	PRPH2	6p21.1	Outer segment membrane structure
17	RDH12	14q24.1	A cause of Leber congenital amaurosis type 13 and Retinitis Pigmentosa 53
18	RHO	3q22.1	Phototransduction, outer segment membrane structure
19	ROM1	11q12.3	Compaction of outer segment disks or in the maintenance of the curvature of the rim
20	RP1	8q12.1	Synergistic roles in affecting photosensitivity and outer segment morphogenesis of rod photoreceptors
21	RP9	7p14.3	This protein localizes in nuclear speckles containing the splicing factors, and has a role in pre-mRNA splicing
22	RPE65	1p31.2	Produced in a thin layer of cells at the back of the eye called the retinal pigment epithelium (RPE).
23	SEMA4A	1q22	Semaphorins are involved in axon guidance, morphogenesis, carcinogenesis, and immunomodulation
24	SNRNP200	2q11.2	Specialized RNA and protein subunits that removes introns from a transcribed pre-mRNA segment
25	TOPORS	9p21.1	It is highly expressed in the testis, and functions as an ubiquitin-protein E3 ligase

Symptoms and diagnosis

The longer to adjust to the darkness is the first symptom because rod cells are affected first also known as night blindness. There is also a loss of peripheral vision at night, as well as a reduction in eyesight and tunnel vision. When you can't see items to the side without rotating your head, you have tunnel vision. Cones are frequently impacted later in the disease's progression. There is photophobia, which is an excessive sensitivity to light, and it may be difficult to see in strong lighting since the cone cells die first. A thorough history, as well as a neurologic and neuro-ophthalmic examination that takes into account visual fields, are required for the treatment of photophobia; it is a condition that causes bursts of light to shimmer or flicker [23]. The majority of the symptoms manifest themselves in early adulthood or youth. The most visible sign of the condition is loss of vision. Because progressive RP is gradual, most people never lose their vision entirely. Night blindness, loss of peripheral vision, difficulty adapting to changes in light or dim light, and difficulty seeing in bad weather are the most common symptoms. The existence of peripheral vision reduces the size of the tunnel vision. Colors such as green and blue are difficult to perceive. Patients with RP have clumsiness as well as visual loss, which can be partial or full. Cataracts with fuzzy vision are one of the symptoms of the illness that might create problems in the latter stages of RP. Visual field testing is used to assess peripheral vision, visual acuity measures how well you can see progressively smaller objects, a dark adaptometery test measures how well you can see in the dark, and the ERG measures the electrical response of the retina to a light stimulus [24].

The initial indication of the condition is trouble seeing in bright or low light, as well as poor night vision. People suffering from RP also lose their visual acuity and side vision. Tunnel vision occurs when the outer vision is dark and the center of vision is contracting. An ophthalmoscope is required to check the illness since it allows the physician to view into the eye via the pupils. The orange or orange-red fundus is observed by the physician in normal eyes. The appearance of black or dark-brown spots in the fundus indicates that the person has RP. The ERG is used to confirm the diagnosis of RP; in this test, lights of various colors and intensity levels are scarcely flashed into the patient's eyes through an enormous reflecting globe. Ensure the proper functioning of the photoreceptors in the retina since there is less electric activity in that area [25]. Ocular diseases or prechiasmal optic neuropathy within the visual pathway are commonly responsible for monocular or binocular visual impairment. RP symptoms include difficulties seeing details, glares, reading problems, restricted peripheral vision, and poor night vision. To assess the degree of the eyesight loss. Visual tests can be performed to determine the disease's genetic mutation [26].

Retinal rods are damaged, resulting in poor night vision that generally begins in maturity but can occur as early as childhood. The development of the ailment is early in the severe types, whereas it may occur in the fifth or sixth decades of life in the lesser variants. The narrowing peripheral vision loss is followed by tunnel vision. The condition causes a ring pattern of vision loss apparent by visual field tests, in which the peripheral ring scotoma gradually spreads, and central vision may be compromised as well. Furthermore, macular dystrophy becomes more active and can lead to blindness; patients suffer from RP report of little shimmering of flickering lights and flashes of light. Hearing loss is one of the symptoms of Usher syndrome and is related to RP in certain people. Funduscopy, in conjunction with electroretinography, is used to diagnose suspected patients with poor night vision [27].

Treatment

In terms of treatment for RP, the ERG, which shows how the retina responds to vision and optical coherence tomography are used. Light mobility is used in tomography to provide detailed pictures of the retina. The technique is known as fundus autofluorescence imaging, and it involves the physician photographing the retina using blue light. The heterogeneity of RP determines the vast range of onset of symptoms. The importance of the Luxturna® results may be seen in the increased number of gene-based clinical research focusing on specific retinal degenerative diseases. The formal treatment is given to the patient; symptoms frequently intensify as they develop to the advanced stage. Luxturna is now the sole approved treatment for RP. It is an approved medication for people with mutations to overcome RPE65 deficiency. Vitamin supplements, protection from visible radiation, and visual assistance can provide the greatest supportive care. Night blindness caused by RP symptoms can be treated with vitamin A supplementation. RPE65 is required for the production of 11-cis-vitamin A during the retinal visual cycle. There is presently no cure or effective medication available to delay or stop the progression of the condition [28].

In more severe instances, the therapy is primarily focused on preventing cone photoreceptor degeneration, which prevents blindness, as well as vision improvement, photophobia reduction, and peripheral vision improvement. Inhibiting the visual cycle is a currently under-investigation strategy for treating retinal degenerative diseases. Furthermore, the night blindness associated with this medication may significantly impair patients' ability to drive at night, among other serious problems [29]. Clinical trials for the novel therapy are currently underway, and persons with RP are receiving auto serum injections. The vision rehabilitation procedure and occupational therapy may aid in illness healing. In pharmaceutical innovations, voretigene neparvovec is only accessible for a small percentage of RP patients with the particular RPE65 mutation; there is no viable curative treatment for the vast majority of RP patients, with significant unmet demand. Formal therapies, such as vitamin A supplements, visual assistance, sun protection, and surgical procedures, may contribute to delaying the progression and alleviating the symptoms of RP. Vitamin A and vitamin E supplementation have been demonstrated to be useful. Night blindness and tunnel vision are symptoms that can be cured with devices such as magnifying glasses and infrared night vision. Although ultraviolet radiation might aggravate the illness, wearing sunglasses can help to protect it [30].

The correct method of capturing history can provide information on the type of rapid representation. Genetic counseling and testing can determine a child's risk for the condition. The advancements made to reduce the risk of this condition have been based on a better knowledge of the pathophysiological pathways and scientific developments in these disorders [31]. Gene therapy methods for dominantly inherited disorders may face more challenging obstacles. With gene therapy and stem cell technology research into the genes causing RP and in upcoming clinical trials, there is hope for treatment for inherited RP. Stem cell transplantation-based approaches have had some success in recent years, particularly the transplantation of bone marrow-derived mesenchymal stem cells into retinal cells and the integration of cells to replace apoptotic or injured retinal cells. There is encouraging progress in clinical trials where marrow-derived mononuclear cells were injected into the vitreous of RP patients. Together, technological advances in gene and cell therapy, promising early-stage human research outcomes, and possible crucial trials suggest that some of these previously unimaginable procedures are now a predictable, near-term reality [32]. Summary of all the articles included in the review is listed in Table 3.

**Table 3:** Summary of all the articles included in the review

Authors	Year	Country	Findings
Poornachandra et al., [1]	2022	India	Patients with retinal dystrophies may benefit from improved care in the future, as new genetic diagnostic techniques and clinical studies show encouraging results.
Kannabiran C, [2]	2019	Singapore	The rod and cone-dominated diseases are classified as either stationary or progressing. These disorders are further grouped based on their manner of inheritance.
Fahim A, [3]	2018	USA	Recent developments in imaging technologies, DNA sequencing, gene therapy, and stem cell biology are currently under investigation and stem cell therapeutics.
Ferrari et al., [5]	2011	Italy	The genetics of RP is complicated by the vast variation of the condition. It is discussed which genes are implicated in the formation of RP and how mutations may lead to retinal degeneration.
Michaelides et al., [6]	2003	UK	Enhanced understanding of the mechanics of hereditary macular dystrophy, which aided in the refinement of diagnosis, illness categorization, and prognosis, as well as enhanced genetic counselling
Prelich G, [8]	2012	USA	The many mechanisms of overexpression employed for reagents accessible in numerous species, as well as the significance of overexpression to human illness, are discussed.
Tolone et al., [9]	2019	Europe	It discusses how major development efforts would go into translating new drugs or biomarkers based on cGMP signaling, along with the drug delivery technology that goes with them.
Megaw et al., [10]	2015	USA	Key models used to test and treat RPGR illness and imply that gene augmentation treatment. Cell replacement treatment based on retinal progenitor cells is a longer-term promise claim.
Veltel et al., [11]	2009	UK	RPGR and RP2, the two gene products, were discussed, and an effort was made to link the genetic information of the patients with the functional information of the corresponding proteins.
Lu et al., [12]	2021	USA	Vascular alterations were shown to be significantly affected by OCTA in contrast to healthy controls. The abnormalities were associated with both choroidal atrophy and loss of central vision.
Scalabrino et al., [13]	2022	UK	Cone and rod medicine are examined because gene remedies for rod degenerative illnesses are likely to prolong cone-mediated vision even in the event that cone shape and density change.
Lang et al., [14]	2019	USA	The onset of RP has been connected to vascular abnormalities. Although PR degeneration is not primarily caused by vascular abnormalities in the fovea and parafovea
Kukreja et al., [15]	2012	USA	There is a discussion on the significance of the cGMP pathway. A key intracellular second messenger that controls a variety of tissue and cellular reactions.
Rivolta et al., [16]	2002	USA	Clinically and genetically, retinitis pigmentosa (RP) and related disorders differ. A large number of genes connected to various diseases are addressed.
Natarajan S, [17]	2011	USA	Concerns have been expressed concerning the possible repercussions of injecting a virus into the eye, as well as the safety of vectors.
Becherucci et al., [18]	2023	USA	The expanding number of current clinical trials investigating the feasibility and effectiveness of various strategies in treating RP heralds a new era in the treatment of uncommon disorders.
Daiger et al., [19]	2013	USA	Existing strategies for RP gene identification and mutation detection risks and outstanding issues.
Wright et al., [20]	2010	USA	The genetic and mechanical causes of PR cell death-induced retinal degeneration.
Moiseyev et al., [21]	2005	USA	The discovery of RPE65's isomerohydrolase fills a critical gap for understanding of the visual cycle.
Hamel CP, [22]	2007	USA	Management seeks to delay the degenerative process, cure problems, and assist patients in coping with the social and psychological consequences of blindness.
Digre and Brennan, [23]	2012	USA	Clinical features and diseases related with photophobia, the anatomy and physiology of this condition, and a practical approach to diagnosis and therapy.
Hartong et al., [24]	2006	USA	The results of controlled studies of dietary interventions to decrease illnesses, Newly discovered genes, research, and urgent cures are all greatly awaited.
Sugawara et al., [25]	2010	USA	The significant relationship between peripheral visual field loss and vision-related quality of life score determines the degree of visual field loss.
Raharja and Whitefield, [26]	2022	USA	A thorough first investigation is essential in determining the urgency of the recommendation.
Muniz et al., [27]	2007	USA	Underlines the importance of understanding how cone photoreceptors maintain ability to detect light
Maguire et al., [28]	2021	USA	Considerations for Luxturna administration were discussed, as well as how the Luxturna experience may lead to future gene-based therapeutics for blindness.
Palczewski K, [29]	2010	USA	Pharmacological vision recovery holds great potential for the development of improved therapies for those who are on the verge of blindness or have lost all of their sight.
Wu et al., [30]	2023	USA	This included categorization, epidemiology, clinical symptoms, prognosis, and traditional treatments.
Sahel et alo., [31]	2014	USA	Progress toward IRD therapeutic strategies, as well as advancements in the pathophysiological processes and technology breakthroughs.
MacLaren et al., [32]	2016	USA	Stem cell-derived transplants were evaluated to relieve, reverse, and restore disease processes.

## Conclusions

This comprehensive review investigates the complexities of retinitis pigmentosa (RP), an inherited retinal disorder. The article explores the pathogenesis, symptoms, diagnosis, and treatment options associated with RP. It discusses the genetic heterogeneity of the disease, the diagnostic methods like clinical symptoms, the functional test used to identify it, and the current treatment approaches, including gene therapy. While RP remains a challenging condition with no known cure, advancements in research, such as Luxturna® and ongoing clinical trials, offer hope for potential treatments. The preliminary findings are favorable, and we anticipate great growth in the coming years. Understanding the multifaceted nature of RP, from its genetic origins to clinical manifestations, is critical for both medical professionals and patients. For therapy, researchers examined the condition and developed visual aids, sunglasses to reduce contact with UV radiation, and dorzolamide or acetazolamide eye drops, as well as vitamin A and E supplements.

As further studies and innovations unfold, the quest to mitigate the impact of this condition continues, and individuals affected by RP can find some solace in the ongoing pursuit of effective treatments. This article serves as a foundation for grasping the intricate landscape of RP and highlights the importance of ongoing research to improve the lives of those living with this condition.
